# Autologous platelet-rich gel activated by calcium gluconate for postoperative endometrial repair in moderate-to-severe intrauterine adhesion: a retrospective preliminary evaluation of efficacy

**DOI:** 10.3389/fmed.2026.1865604

**Published:** 2026-10-29

**Authors:** Yunying Li, Yingxue Han, Junjuan Cao

**Affiliations:** 1 Department of Gynecology and Obstetrics, Shijiazhuang People’s Hospital, Shijiazhuang, China; 2 Transfusion Department, Shijiazhuang People’s Hospital, Shijiazhuang, China

**Keywords:** autologous platelet-rich gel, calcium gluconate, intrauterine adhesion, menstruation, recurrence

## Abstract

**Background:**

This study aimed to investigate the effect of calcium gluconate–activated autologous platelet-rich gel (APG) on endometrial repair in patients with moderate-to-severe intrauterine adhesion (IUA).

**Methods:**

In this study, 60 patients with moderate-to-severe IUA who were undergoing hysteroscopic transcervical resection of adhesion surgery were recruited and grouped based on whether they underwent APG treatment (APG group) or not (non-APG group; *n* = 30 each). Hysteroscopy examination and transvaginal ultrasonography were performed after surgery to evaluate adhesion recurrence and endometrial thickness. Simultaneously, patients self-recorded the menstrual volume using a menstrual diary.

**Results:**

There was no significant difference in baseline characteristics, preoperative uterine adhesion grade, endometrial thickness, or menstrual pattern between the APG and non-APG groups (*p* > 0.05). After treatment, compared with the non-APG group, the postoperative adhesion recurrence rate (13.3% vs. 36.7%) and adhesion grades (75% mild and 25% moderate vs. 72.7% mild and 27.3% moderate) were significantly decreased in the APG group (*p* = 0.039). The endometrium thickness in the APG group was greater than that in the non-APG group (6.29 ± 0.24 mm vs. 5.10 ± 0.16 mm, *p* = 0.000), and the endometrial thickness increment was substantially higher in the APG group than that in the non-APG group (2.82 ± 0.17 mm vs. 1.84 ± 0.12 mm, *p* = 0.000). Furthermore, more participants in the APG group experienced significant recovery in menstrual volume at 3 months after surgery (*p* = 0.031), and participants from the APG group maintained improved menstrual volume 6 months after surgery (*p* = 0.029). Multivariate analysis confirmed the beneficial effect of APG in promoting endometrial repair.

**Conclusion:**

Calcium gluconate–activated autologous platelet-rich gel has the potential to reduce postoperative recurrence and improve menstrual properties in patients with moderate-to-severe IUA.

## 1 Introduction

Intrauterine adhesions (IUAs) can lead to reduced menstrual volume, shorter menstrual periods, amenorrhea, infertility, and miscarriage. Common causes of IUAs include infections and various intrauterine procedures, such as induced abortion, dilatation and curettage (D&C), and the introduction or removal of intrauterine contraceptive devices (IUDs) (1). Such events can damage the basal layer of the endometrium, leading to disordered cell generation and delayed wound healing. Moreover, exposure of the uterine muscle layer and disruption of the microenvironment ultimately result in partial or complete closure of the uterine cavity (2).

Hysteroscopic transcervical resection of adhesions (TCRA) surgery is currently the preferred method for IUA treatment. TCRA can improve the morphology of the uterine cavity; however, effective prevention of adhesion reformation is also a key to treatment, and the prevalence of severe postoperative IUA recurrence can reach 62.5% (3). Conventional methods for the prevention of adhesion recurrence include hormone therapy, the placement of a balloon or an IUD as a mechanical barrier, and antiadhesion agents such as sodium hyaluronate (4, 5). These approaches are limited by the need for long treatment cycles, restricted efficacy for moderate-to-severe IUA, and risk of complications (2).

Platelet-rich plasma (PRP) is extracted from the peripheral blood of patients and has a platelet count that is three to five times that of whole blood (6). After activation of PRP, platelets degranulate and release large amounts of growth factors and cytokines, such as platelet-derived growth factors, vascular endothelial growth factor, and epidermal growth factor. PRP promotes cell proliferation, cell migration, and matrix remodeling (7). There is strong evidence that PRP has significant effects in treating adhesive capsulitis (8) and patellar tendinopathy (9) and in promoting wound healing in patients with diabetes (10). In addition, PRP has the potential to increase endometrium thickness and immune tolerance (11). The addition of agonists to PRP leads to the formation of autologous platelet-rich gel (APG), which releases bioactive components slowly and protects them from proteolytic enzyme degradation (12). Furthermore, APG can function comparably to a mechanical barrier.

The purpose of this study was to conduct a preliminary evaluation of the efficacy of calcium gluconate–activated APG in the prevention of recurrence and promotion of menstrual recovery in patients with moderate-to-severe IUA after surgery.

## 2 Materials and methods

### 2.1 Patient recruitment

This retrospective cohort study was conducted between 2022 and 2025 at the Shijiazhuang People's Hospital. The hospital’s ethics committee approved this study. Clinical records of 350 patients who underwent hysteroscopic TCRA surgery for moderate-to-severe IUA were reviewed. Among them, 107 patients completed postoperative treatment and follow-up. The inclusion criteria were (1) healthy women aged 20–40 years, (2) patients diagnosed with moderate-to-severe IUA through hysteroscopy according to the American Fertility Society (AFS) intrauterine score system (mild, 1–4; moderate, 5–8; and severe, 9–12) (13), (3) patients with normal hormonal evaluation, and (4) patients without contraindications for the use of hormone drugs. The exclusion criteria were (1) patients with a hysteroscopic TCRA history; (2) patients with uterine lesions, such as uterine malformations, endometrial polyps, uterine myomas, and inflammation; (3) patients with tumors or systemic diseases, such as cardiovascular diseases, respiratory diseases, tuberculosis, or immune system diseases; (4) patients who had not been treated according to the prescribed plan; (5) missing follow-up or incomplete information; and (6) patients who had undergone other treatments with potential to influence the research results, such as traditional Chinese medicine and balloon catheters. According to these criteria, 60 patients were included.

### 2.2 PRP extraction and freeze-thawing

For each patient, PRP was prepared using a fully automated blood cell separator (NGL XCF 3000, Nigale Biomedical, Sichuan, China) under strict, sterile, and quality-controlled conditions. A venous puncture was performed after standard skin disinfection. All disposable kits were sterilized using ethylene oxide and checked for package integrity and expiry date before use, and operators wore sterile gowns, caps, masks, and gloves throughout the procedure. The separation process adopted a closed fluid circuit to avoid exposure of blood components to non-sterile surroundings. Whole blood and the final PRP product were sent for complete blood count analysis. The platelet concentration in whole blood was 245 ± 85 × 10^9^ platelets/L. Quality indicators included platelet concentration (1,200 ± 420 × 10^9^TCRAplatelets/L), platelet enrichment (four to six fold), residual red blood cell count (0.05 ± 0.04 × 10^12^/L), and white blood cell count (0.8 ± 0.1 × 10^9^/L). Each patient received autologous PRP prepared individually. Approximately 30 mL of PRP was extracted using a blood cell separator, and the remaining blood components were returned back to the patient. The prepared PRP was divided into an average of five portions and stored in five separate freezing bags, which were kept frozen at −80 ℃ (14). At the appropriate time, a PRP freezing bag was thawed in a plasma thawer (KJX-IA, Suzhou, China) and used within 30 min. Each PRP freezing bag could be frozen and thawed only once.

### 2.3 Hysteroscopic TCRA surgery

After anesthesia induction, each patient was placed with a disposable cervical dilator (Shuangji, Zhejiang, China) composed of a polymer material that absorbs liquid and swells. Anesthesia was maintained throughout the interval between cervical dilation and the initiation of the hysteroscopic procedure. Thirty minutes later, the dilator was removed and a hysteroscope (outer body diameter ≤4 mm, inner tube sheath diameter ≤8 mm, and outer tube sheath diameter ≤8.5 mm/26 Fr; Karl Storz, Tuttlingen, Germany) was inserted into the uterine cavity. A sodium chloride (0.9%) injection served as the surgical medium. During the procedure, the pressure of the uterine cavity was lower than the mean arterial pressure of the paper, and a bipolar plasma needle electrode (Karl Storz, Tuttlingen, Germany) was used to separate adhesions.

### 2.4 APG intrauterine infusion

A disposable hysterosalpingography tube (Macklin, Suzhou, China) was inserted into the uterine cavity of each patient, and the balloon was filled with 1–2 mL of gas to seal the cervix. Ten percent calcium gluconate was then added to PRP at a 1:9 ratio and was mixed well (15). After mixing for 5–15 min (before the formation of APG), approximately 5 mL of the mixed solution was infused into the uterine cavity using a non-resistance perfusion method. Patients were asked to maintain their position for 15–20 min; then, the gas was discharged; and the tube was slowly removed. The first intrauterine infusion was performed immediately after hysteroscopic TCRA surgery on the same day. Subsequently, patients underwent APG treatment every 7–10 days in the outpatient operating room without anesthesia, and they did not express any obvious discomfort. Patients who had a menstrual cycle underwent APG intrauterine infusion on the first and eighth days after the cessation of menstruation. When the endometrium thickness reached ≥7 mm, the operation was stopped to avoid the risk of endometriosis caused by intrauterine infusion.

### 2.5 Routine treatment and follow-up

Patients were routinely administered periodic treatment with estrogen (estradiol valerate; Bayer, Leverkusen, Germany; 4 mg daily for 21 days) and progesterone (dydrogesterone; Duphaston, Abbott, Netherlands; 20 mg daily for the last 10 days) during the study. Patients chose whether to undergo APG intrauterine infusion based on treatment intention and their economic situation. Patients in the APG group received an intrauterine infusion of APG alongside hormone therapy for 3 months, and those in the non-APG group were given hormone therapy alone. The number of intrauterine infusions in the APG group ranged from 6 to 10. Hysteroscopic examination was performed 6–8 weeks after hysteroscopic TCRA surgery to determine whether adhesions had recurred postoperatively. If IUAs were found, further surgery was performed to separate them. All assessments of hysteroscopic adhesion adopted the AFS score system. Transvaginal ultrasonography GE Voluson E10 (GE Healthcare, Milwaukee, WI, USA) was performed by a single experienced gynecologic ultrasonographer 3 months after the hysteroscopic TCRA surgery during the mid-luteal phase. Double-layer endometrial thickness was measured on the maximum sagittal plane of the uterus at the thickest endometrial site. One stable measurement was documented for each participant. The ultrasonographer was not blinded to group assignment. Changes in menstrual volume 3 and 6 months after surgery were recorded based on patient menstrual diaries (Figure 1).

**Figure 1:**
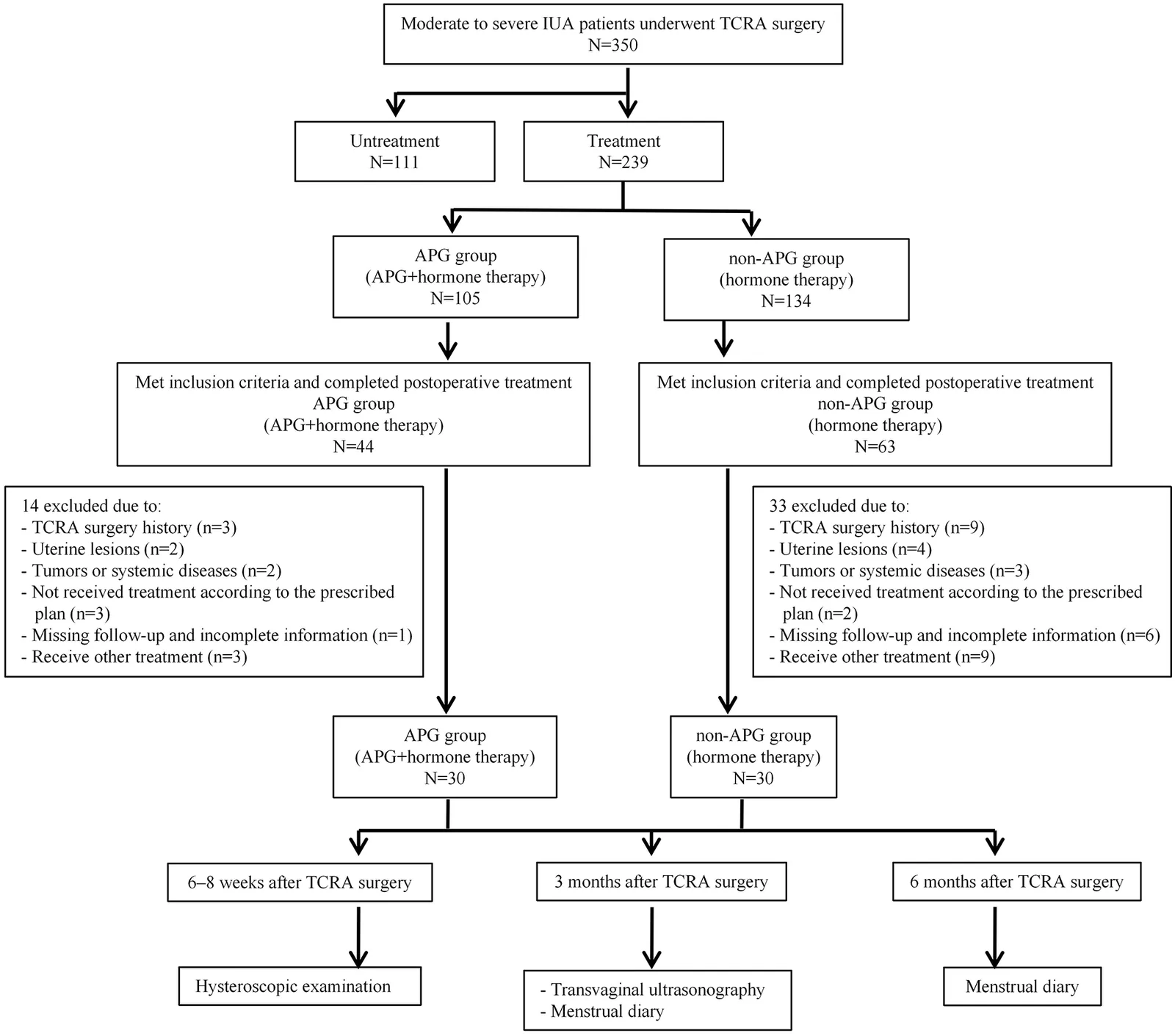
A flowchart of the study.

### 2.6 Statistical analysis

Data analysis was conducted using SPSS 21.0 software (IBM, Inc., Armonk, NY, USA). Data are summarized as mean ± SE or percentages. Student's t-test was used to compare age, BMI, and endometrial thickness between the two groups, while chi-square test was applied to compare parity, D&C, the history of hysteroscopic surgery, adhesion grades, and the menstrual pattern. In addition to unadjusted analyses, ordinal logistic regression analysis was used to explore the effect of APG on postoperative adhesion severity. Postoperative adhesion severity was treated as an ordinal categorical variable (no adhesion, mild, moderate, or severe), while the baseline AFS score was included as a continuous covariate. Inverse probability of treatment weighting was adopted as a sensitivity analysis, and standardized mean differences were calculated to assess covariate balance. Multivariable linear regression was applied for continuous outcomes, and ordinal logistic regression was applied for menstrual outcomes. The proportional odds assumption was evaluated using the nominal test. Postoperative endometrial thickness at 3 months was set as the primary outcome. Secondary outcomes included increment in endometrial thickness, postoperative adhesion severity, menstrual recovery at 3 months, and menstrual maintenance at 6 months, which were analyzed by analysis of covariance (ANCOVA). Secondary outcome analyses were corrected using Holm–Bonferroni and Benjamini–Hochberg false discovery rate (FDR) methods. A two-sided *p*-value <0.05 was considered statistically significant. Graphs were created using Prism 8.0 software (GraphPad Prism, San Diego, CA, USA).

## 3 Results

### 3.1 Participant baseline characteristics

Of the 60 participants, 30 were assigned to the APG and 30 to non-APG groups. There were no differences in age, BMI, parity, prevalence of D&C, or the history of hysteroscopic surgery between the two groups (*p* > 0.05; Table 1).

**Table 1:** Baseline characteristics of the participants.

Variables	APG group (*n* = 30)	Non-APG group (*n* = 30)	*p-*value
Age (year)	34.00 ± 0.48	33.63 ± 0.47	0.173
BMI (kg/m^2^)	22.32 ± 0.33	21.47 ± 0.33	0.074
Number of parturitions			
0	7 (23.3%)	6 (20%)	0.269
1	9 (30%)	6 (20%)	
2	12 (40%)	13 (43.3%)	
>2	2 (6.7%)	5 (16.7%)	
D&C			
1	5 (16.7%)	8 (26.7%)	0.669
2	13 (43.3%)	10 (33.3%)	
>2	12 (40%)	12 (40%)	
History of hysteroscopic surgery			
Yes	9 (30%)	8 (26.7%)	0.776
No	21 (70%)	22 (73.3%)	

APG, autologous platelet-rich gel; BMI, body mass index; D&C, dilatation and curettage.

Data were given as mean ± standard error of the mean or *n* (%).

a *p* < 0.05 was considered statistically significant in the comparison of the APG group with the non-APG group.

### 3.2 Postoperative APG intrauterine infusion reduced adhesion recurrence and increased the endometrial thickness

As shown in Table 2, preoperative adhesion grades did not differ significantly between the APG and non-APG groups. Figure 2 presents comparative images pre- and posthysteroscopic TCRA surgery. The postoperative prevalence of adhesion recurrence (13.3% vs. 36.7%) and adhesion grades (75% mild and 25% moderate vs. 72.7% mild and 27.3% moderate) were significantly lower in the APG group than in the non-APG group (*p* = 0.039). After treatment, the endometrial thickness was greater in the APG group (6.29 ± 0.24 mm) than in the non-APG group [5.10 ± 0.16 mm; mean difference = 1.183, 95% confidence interval (CI): 0.608–1.759, and *p* = 0.000]. Furthermore, the endometrial thickness increment was higher in the APG group (2.82 ± 0.17 mm) than in the non-APG group (1.84 ± 0.12 mm; mean difference = 0.983, 95% CI: 0.566–1.400, and *p* = 0.000; Figures 3, 4).

**Table 2:** Comparison of the recurrence of adhesions between two groups after treatment.

Variables	APG group (*n* = 30)	Non-APG group (*n* = 30)	*p-*value
Preoperative adhesion grades			
Mild	0 (0%)	0 (0%)	0.288
Moderate	21 (70%)	17 (56.7%)	
Severe	9 (30%)	13 (43.3%)	
Postoperative adhesion grades			
No adhesion	26 (86.7%)	19 (63.3%)	0.039^a^
Mild	3 (10%)	8 (26.7%)	
Moderate	1 (3.3%)	3 (10%)	
Severe	0 (0%)	0 (0%)	

Data were given in *n* (%).

a *p* < 0.05 was considered statistically significant in the comparison between the APG group and the non-APG group.

**Figure 2:**
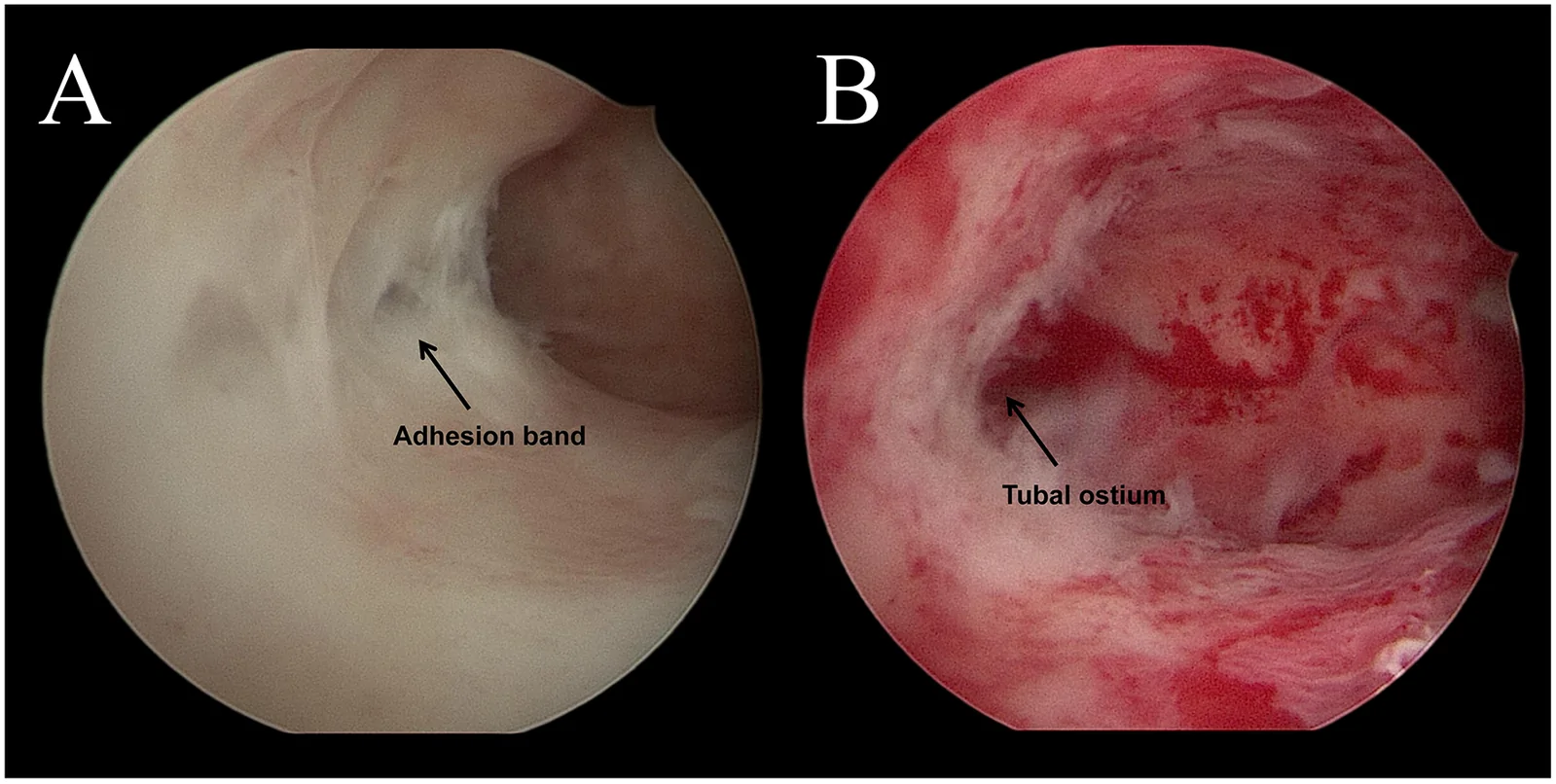
A comparative image of preoperative and postoperative hysteroscopic TCRA surgery. **(A)** A hysteroscopic image taken before surgery showing moderate-to-severe intrauterine adhesions (AFS grade III). **(B)** An image of the immediate postoperative cavity in which the tubal ostium is visible.

**Figure 3:**
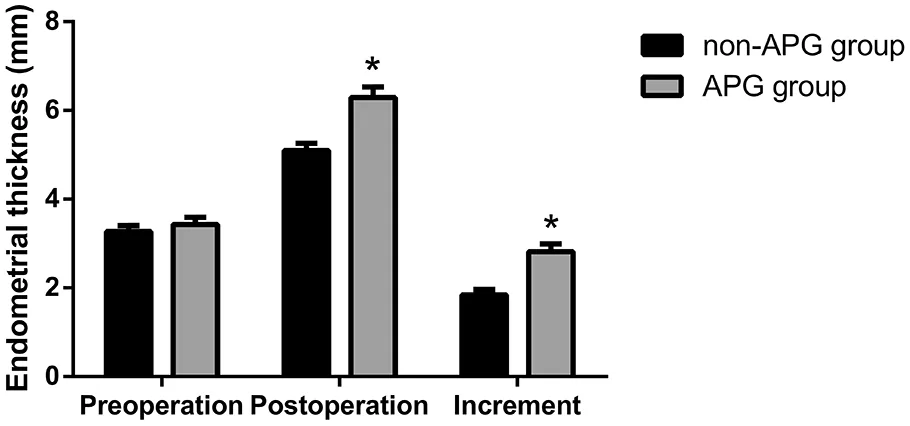
The thickness of the endometrium in the APG group was significantly increased compared with that in the non-APG group. Data were presented as mean ± standard error of the mean (SEM; in millimeters). The asterisks indicate statistically significant differences between groups (*p* < 0.05).

**Figure 4:**
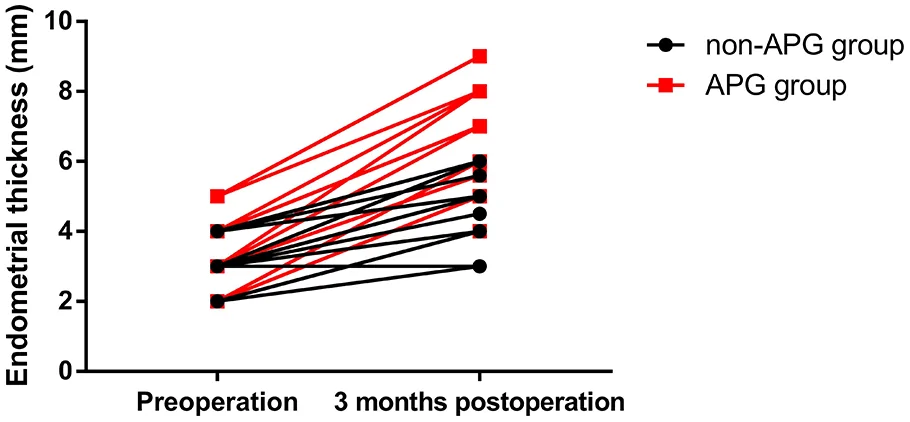
A spaghetti plot of individual changes in endometrial thickness before surgery and at 3 months postoperation in the APG and non-APG groups.

After adjustment for the baseline AFS score and preoperative endometrial thickness using ordinal logistic regression, the postoperative adhesion severity was significantly lower in the APG group than in the non-APG group (OR = 0.252, 95% CI: 0.057–0.929, and *p* = 0.038). Further adjustment for the number of D&C yielded consistent results (OR = 0.252, 95% CI: 0.056–0.931, and *p* = 0.038). The proportional odds assumption was not violated (nominal test: group *p* = 0.762 and pre-AFS *p* = 0.638). Effective sample sizes after weighting were 27.05 and 21.24 for the APG and non-APG groups, respectively; no extreme weights were observed. Weighted ordinal logistic regression demonstrated milder postoperative adhesion severity in the APG group, with an effect direction consistent with the primary analysis (OR = 0.286, 95% CI: 0.070–1.160, and *p* = 0.085), although the result did not reach statistical significance. This finding may be attributable to the reduction in the effective sample size after weighting (Table 3).

**Table 3:** Postoperative adhesion grade: unadjusted and adjusted analyses.

Analysis	Comparison	OR (95% CI)	*p-*value
Unadjusted (chi-square)	APG vs. non-APG	—	0.039
Ordinal logistic, model 1	APG vs. non-APG	0.262 (0.061-0.954)	0.052
Ordinal logistic, model 2	APG vs. non-APG	0.252 (0.057–0.929)	0.038
Ordinal logistic, model 3	APG vs. non-APG	0.252 (0.056–0.931)	0.038
IPTW-weighted ordinal logistic	APG vs. non-APG	0.286 (0.070–1.160)	0.085

Model 1: group + baseline AFS score (continuous). Model 2: model 1 + preoperative endometrial thickness. Model 3: model 2 + number of D&C procedures. IPTW: the propensity score model included the age, BMI, baseline AFS score, preoperative endometrial thickness, D&C, hysteroscopic surgery history, and preoperative menstrual status; all 60 patients were retained. The postoperative adhesion grade was treated as an ordered categorical variable (none, mild, moderate, and severe). OR < 1 indicates a lower (better) postoperative adhesion grade in the APG group. The proportional odds assumption was satisfied (nominal test: group *p* = 0.762; pre_AFS *p* = 0.638). In the IPTW-weighted analysis, the effective sample sizes were 27.05 (APG) and 21.24 (non-APG).

### 3.3 Postoperative APG intrauterine infusion improved menstrual volume in patients with IUA

More patients in the APG group than the non-APG group experienced a significant recovery in menstrual volume 3 months after surgery (*p* = 0.031). In addition, fewer patients in the APG group had a low menstrual volume 6 months after surgery (*p* = 0.029; Table 4).

**Table 4:** Comparison of the improvements in menstruation of the two groups after treatment.

Variables	APG group (*n* = 30)	Non-APG group (*n* = 30)	*p-*value
Menstrual pattern (preoperation)			
Normal	0 (0%)	0 (0%)	0.792
Hypomenorrhea	18 (60%)	19 (63.3%)	
Amenorrhea	12 (40%)	11 (36.7%)	
Menstruation recovery situation (3 months after surgery)			
Mild recovery	0 (0%)	0 (0%)	0.031^a^
Moderate recovery	3 (10%)	5 (16.7%)	
Substantial recovery	7 (23.3%)	14 (46.7%)	
Significant recovery	20 (66.7%)	11 (36.7%)	
Menstruation maintenance situation (6 months after surgery)			
No change	25 (83.3%)	17 (56.7%)	0.029^a^
Mild reduce	4 (13.3%)	11 (36.7%)	
Severe reduce	1 (3.3%)	2 (6.6%)	

Data were given in *n* (%). Slight recovery, less than 1/3 of the normal menstrual volume; moderate recovery, 1/3–1/2 of the normal menstrual volume; substantial recovery, 1/2–2/3 of the normal menstrual volume; and significant recovery, more than 2/3 of the normal menstrual volume. The menstruation maintenance situation indicates the changes in menstrual volume at 6 months after surgery compared with those at 3 months after surgery—mild reduction: a decrease in menstrual volume of less than 1/2; and severe reduction: a decrease in menstrual volume of more than 1/2.

a *p* < 0.05 was considered statistically significant in the comparison of the APG group with the non-APG group.

### 3.4 Primary and secondary outcome analysis

Postoperative endometrial thickness at 3 months was defined as the primary outcome. After adjustment for preoperative endometrial thickness and baseline AFS score, the postoperative endometrial thickness at 3 months was significantly higher in the APG group than in the non-APG group (*β* = 0.95, 95% CI: 0.54–1.37, and *p* < 0.001). *p*-values for secondary outcomes were corrected using the Holm–Bonferroni method, and Benjamini–Hochberg FDR-corrected results are also reported. The endometrial thickness increment remained significant after correction (adjusted *p* = 0.0001). The postoperative adhesion severity, menstrual recovery at 3 months, and menstrual maintenance at 6 months did not reach statistical significance after Holm correction (adjusted *p* = 0.098, 0.098, and 0.094, respectively), whereas all *p-*values were 0.050 following Benjamini–Hochberg FDR correction (Table 5).

**Table 5:** Adjusted analyses for endometrial thickness and menstrual outcomes with multiplicity correction.

Outcome	Model	Effect estimate (95% CI)	Raw *p*	Holm-adjusted *p*	BH-adjusted *p*
Postoperative EMT at 3 months (mm; primary)	Group + pre_EMT + pre_AFS	*β* = 0.95 (0.54–1.37)	<0.001	—	—
EMT increment (mm)	Group + pre_EMT + pre_AFS	*β* = 0.95 (0.54–1.37)	<0.001	0.0001	0.0001
Adhesion grade	Group + pre_AFS + pre_EMT	OR = 0.252 (0.057–0.929)	0.049	0.098	0.050
Menstrual recovery at 3 months	Group + pre_AFS + pre_menstrual	OR = 2.96 (1.02–9.12)	0.050	0.098	0.050
Menstrual maintenance at 6 months	Group + pre_AFS + pre_menstrual	OR = 0.257 (0.068–0.842)	0.031	0.094	0.050

EMT, endometrial thickness; pre_EMT, preoperative endometrial thickness; pre_AFS, baseline American Fertility Society score.

The primary outcome was postoperative EMT at 3 months and was not adjusted for multiplicity. Secondary outcomes were adjusted using the Holm–Bonferroni method, with Benjamini–Hochberg FDR results also reported. A two-sided *p*-value <0.05 was considered statistically significant.

## 4 Discussion

IUA is a common gynecological disease that principally develops because of a deficiency in endometrial cell repair after injury. Patients with IUA often seek medical advice due to hypomenorrhea, amenorrhea, and infertility. For patients with IUA who desire future pregnancy, delayed diagnosis or insufficient treatment can lead to irreversible endometrial damage, ultimately compromising fertility outcomes. Hysteroscopic TCRA surgery, which improves the uterine cavity morphology, is the preferred method of treatment for such patients; however, the prevalence of postoperative adhesion recurrence is high. Recent reports have highlighted the need to enhance the competence of physicians through hysteroscopic TCRA-related training programs (16). The restoration of normal endometrial function is as crucial as anatomical reconstruction. Therefore, the implementation of early screening and effective endometrial repair strategies are of particular importance in this subpopulation.

In recent years, PRP, a cellular therapy used in regenerative medicine, has become the focus of increasing attention and the subject of a considerable amount of research. PRP promotes cellular proliferation by increasing the expression of cyclin A, CDK1, and CDK2 (17). Clinically, PPR has advantages including easy acquisition, low cost, avoidance of disease transmission, and lack of rejection reactions. PRP has been widely used to treat osteoarthritis (18) and acute and chronic wounds (19). In addition, PRP improves ovarian function, the likelihood of a woman becoming pregnant by reducing follicle-stimulating hormone levels and increasing anti-Müllerian hormone levels, and the number of high-quality embryos produced (20).

PRP improves endometrium thickness and receptivity by increasing angiogenesis and exerting positive effects on immune cells and the microbiota. Consequently, PRP improves menstruation and pregnancy outcomes in patients with a thin endometrium (21) or endometritis (22). In a mouse model of IUA, PRP inhibited cell ferroptosis, autophagy, and pyroptosis by upregulating melanotransferrin and retinol metabolism, thereby accelerating endometrial regeneration (23). However, there have been few clinical studies on the postoperative use of PRP in patients with IUA. Javaheri et al. (24) found no significant differences in menstruation or recurrence of adhesions between patients with IUA who were administered 1 mL of PRP via a single intrauterine injection following surgery and those who were not. Furthermore, Aghajanova et al. (25) showed that estrogen combined with 0.5–1 mL of PRP administered via a single intrauterine injection following IUA surgery did not have additional effects on endometrial thickness or the likelihood of becoming pregnant relative to estrogen alone. These findings may be explained by the liquid state of the PRP used in these studies, resulting in the lack of retention in the uterine cavity for sufficient time to exert its effects. Zhang et al. (26) injected PRP into the endometrium of patients with moderate-to-severe IUA under hysteroscopic monitoring and observed reduced adhesion scores, improved menstrual symptoms, and increased clinical pregnancy rates; however, this technology involves an invasive procedure that carries risks, including bleeding, infection, and recurrence, and requires high-specification surgical instruments and skilled physicians.

PRP can be transformed into APG via the addition of an activator. The advantage of the gel form is that bioactive factors can be protected against degradation and released slowly (12). Among common PRP activators, calcium gluconate does not carry the risk of heterogeneity due to thrombin, while its effect in activating cytokines is similar to that of thrombin. The gel formed by calcium salts retracts more slowly. Furthermore, calcium gluconate use is associated with a smaller amount of calcium deposition than with the use of calcium chloride (27). Sills et al. (28) reported that the ovarian function of patients with premature ovarian insufficiency was improved by injection of calcium gluconate–activated PRP gel into the ovaries. Furthermore, during laparoscopic orthotopic reimplantation of cryopreserved ovarian tissue, use of frozen-thawed ovarian tissue fragments covered with the calcium gluconate–activated PRP gel can potentially enhance restoration of ovarian function of patients after surgery (29). In this study, we also chose calcium gluconate as a PRP activator to produce APG. Our report is the first to describe the application of a calcium gluconate–activated APG intrauterine infusion after IUA surgery. We found that, compared with hormone therapy alone, combined APG intrauterine infusion was associated a greater increase in endometrial thickness, higher menstrual volume, and lower recurrence rate and adhesion grade in patients with moderate-to-severe IUA. These preliminary findings suggest that APG intrauterine infusion combined with hormone therapy represents a potentially effective approach for postoperative endometrial repair in patients with IUA.

Given its high recurrence rate and potential long-term impact on reproductive health, primary prevention of IUA should be prioritized (30), including minimizing unnecessary intrauterine manipulations, adopting atraumatic surgical techniques, and implementing early postoperative surveillance in high-risk populations. A shift from a purely therapeutic approach to a combined preventive and therapeutic strategy is warranted to reduce the overall disease burden.

This study was different from previous investigations in that a gel form of APG was used. The gel form was chosen because it can both slow the release of bioactive substances and form a barrier in the uterine cavity; properties that may explain why APG significantly promoted endometrial growth and prevented adhesion formation after the hysteroscopic TCRA surgery. Recent research has shown that hydrogel forms of autologous PRP, including double-network hydrogel (31) and CL-PF127@PRP hydrogel (32), have good biocompatibility, anti-inflammatory function, and angiogenic activity, providing promising prospects for clinical prevention and treatment of IUA.

No complications were observed in this investigation, demonstrating the effectiveness and safety of the procedure. Nevertheless, this study has some limitations. First, the sample size is small, and the study is retrospective and non-randomized, which may have introduced selection bias. Menstrual volume was assessed based on the menstrual diary of patients, rather than the employing the validated pictorial blood loss assessment chart (PBAC) method, which may have introduced subjective bias. Moreover, the statistical significance of secondary outcomes was lost following adjusted analyses. Therefore, our findings should be regarded as exploratory and supportive evidence, rather than confirmatory conclusions. The primary outcome (postoperative endometrial thickness at 3 months) remained robust before and after adjustment, representing the key finding of this study. Further validation in larger, randomized controlled trials is warranted. Second, the uterine cavity was perfused every 7–10 days, considering the length of the platelet turnover cycle; however, optimal perfusion conditions require further exploration. Third, considering the cost and the need for multiple follow-up appointments, clinical application was influenced by the economic position and requirements of patients. Fourth, we did not assess pregnancy and live birth rates, and hence, further research in future should focus on the statistical analysis of these outcomes. Finally, *in vivo* and *in vitro* experiments were not performed, and therefore, studies of gene and protein expression are required to provide a theoretical basis underpinning the postoperative use of APG intrauterine infusion in patients with IUA. We present the findings of a preliminary study of the use of APG activated using calcium gluconate for the treatment of patients with IUA. In the future, we will evaluate the long-term effects of this preparation.

## 5 Conclusion

Our findings suggest that, relative to postoperative hormone therapy alone, combination treatment with APG that is activated by calcium gluconate intrauterine infusion may be associated with a lower probability of adhesion recurrence, greater endometrial growth, and improved menstrual volume in patients with moderate-to-severe IUA.

## Data Availability

The original contributions presented in the study are included in the article/Supplementary Material, and further inquiries can be directed to the corresponding author.
